# Effect of cultivation pH on the surface hydrophobicity of *Bacillus subtilis* spores

**DOI:** 10.1186/s13568-017-0458-2

**Published:** 2017-07-27

**Authors:** Elisabeth Eschlbeck, Simon A. W. Bauer, Ulrich Kulozik

**Affiliations:** 1 Chair of Food and Bioprocess Engineering, Technical University of Munich, Weihenstephaner Berg 1, Freising, DE Germany; 2 ZIEL Institute for Food & Health, Weihenstephaner Berg 1, 85354 Freising, Germany

**Keywords:** *Bacillus subtilis*, Submersed spore production, Bioindicator, Hydrogen peroxide, Resistance, Surface hydrophobicity

## Abstract

*Bacillus subtilis* spores are often used as biological indicators (BI) to monitor decontamination processes with gaseous hydrogen peroxide. Results in practical inactivation validation tests, however, vary considerably with no available explanation so far. This study reports on the effect of cultivation pH on spore surface hydrophobicity. Surface hydrophobicity is suspected to have an impact on the decontamination of technical surfaces such as packaging material when gaseous, condensing hydrogen peroxide is applied. It is the aim of this study to examine the impact of different cultivation pH levels on surface hydrophobicity and resistance of *B.* *subtilis* spores. Submersed cultivation of *B.* *subtilis* in bioreactors at controlled conditions with different static pH levels led to contact angles ranged between 50° and 80°, which was analyzed with water on a homogeneous layer of spores on a filter sheet. Resistance of spores was also affected by the cultivation pH. The results show that the culturing conditions during BI production should be controlled to obtain BI with specified characteristics in inactivation validation tests.

## Introduction

In food and pharma industries sterile surfaces for aseptic packaging and clean rooms are often indispensable. The processes to obtain surface sterility are either based on physical or on chemical action. Hydrogen peroxide (H_2_O_2_) is among the most often used disinfectants. H_2_O_2_, mostly a mixture of 35% H_2_O_2_ in water and low amounts of stabilizers, can be applied in its liquid form as immersion bath, as spray or as vapor (Engelhard and Kulozik 2006; Pruß et al. 2012).

To validate decontamination processes, resistant, non-pathogenic test organisms are applied as biological indicators (BI) as the detection of survivors is the only direct method to assess the inactivation result (Block 2001; Sella et al. 2013). Spores of *Bacillus subtilis* (*B.* *subtilis*) and *Bacillus atrophaeus* (*B.* *atrophaeus*) meet the requirements for BI in inactivation processes with high concentrations of H_2_O_2_ (Sella et al. 2012, VDMA 2006).

The mode of action of vaporized H_2_O_2_ depends on time, temperature of the vapor and surface as well as concentration of H_2_O_2_ and H_2_O in the gas with each of those variables affecting each other. For a decontamination process with vaporized H_2_O_2_ and the intentional formation of condensate, even more influencing factors arise (Agalloco and Akers 2013). Therefore, an easy test method to estimate the resistance of BI towards H_2_O_2_ is required. Resistance tests applying liquid H_2_O_2_ with controlled temperature are used as standard tests at practical level to test the resistance of BI (Muranyi et al. 2006; Pruß et al. 2012; Deinhard et al. 2016).

Pruß et al. (2012) investigated the influence of the surface temperature of packaging specimens on the inactivation effect of *Bacillus* spores with gaseous, condensing H_2_O_2_. From their results they postulated that variable inactivation effects may depend on the surface hydrophobicity of the spores applied as BI. This means that, if condensate formation takes place, spores with a low surface hydrophobicity, i.e. good wettability, would preferably induce condensation and would thus be covered by extremely concentrated H_2_O_2_ condensate. In contrast, spores with a lower wettability would be less affected by the condensate formation. Therefore, inactivation was suspected to be less pronounced for hydrophobic BI.

Surface hydrophobicity of microorganisms is often indirectly measured by the water contact angle (Busscher et al. 1984; Mozes and Rouxhet 1987; van Loosdrecht et al. 1987; Seale et al. 2008). Water contact angle of *B.* *subtilis* in its vegetative and spore form depends on the strain and growth phase and can vary between 10° and 59° (Garry et al. 1998; Ahimou et al. 2001; Mozes and Rouxhet 1987). Microorganisms are classified as hydrophilic if their water contact angle is below 20° and as hydrophobic if they show contact angles above 50° (Rijnaarts et al. 1993).

Several authors report that spore resistance to heat or hydrogen peroxide is significantly influenced by the culturing conditions (Leaper 1987; Melly et al. 2002a; Rose et al. 2007; Minh et al. 2008; Baril et al. 2012). Results are inconsistent and the mechanism of inactivation of spores is still incompletely understood. Melly et al. (2002b) state that liquid H_2_O_2_ leads to damage of the peptidoglycan layer or proteinaceous components, which play a key role in spore core expansion. Therefore, water uptake of the spore and germination are impaired. The resistance of the spores cultivated at different temperatures varied, a high cultivation temperature of 48 °C led to increased resistance compared to a cultivation temperature of 22 or 30 °C. Several spore components known to have spore protecting properties such as SASP (small, acid soluble spore proteins), the amount of dipicolinic acid and the composition of spore coat and spore cortex were analyzed. Significant differences were only observed in the coat protein composition. As the coat protein composition changes with sporulation temperature (Melly et al. 2002a), it is likely that the composition of the outermost layer varies as well. For *B.* *subtilis* spores, this outer layer consists of glycoproteins which tightly surround the outer spore coat. This so-called “crust” is responsible for the surface characteristics of *B.* *subtilis* spores (Imamura et al. 2010; McKenney et al. 2010).

Apart from temperature, cultivation pH also influences spore characteristics. However, those studies investigating the influence of cultivation pH conducted cultivation of spores on agar plates where the pH cannot be controlled (Craven 1990; Mazas et al. 1997). Some years ago Rose et al. (2007) performed a study showing the differences in spore characteristics between spores prepared on agar plates and in liquid medium. Before that, due to simplicity lab-scale studies have often been carried out on agar plates. The relatively new concept of submerged cultivation in bioreactors provides options to control influencing factors throughout fermentation such as maintaining a defined static pH throughout the procedure.

Some authors investigated the influence of pH on spore yield (Monteiro et al. 2005; Baril et al. 2012), but its impact on surface hydrophobicity has not been studied so far. Mazas et al. (1997) investigated the influence of culturing pH on the resistance of *B.* *subtilis* spores against heat. They observed a decreasing D_100 °C_ value the lower the cultivation pH was. Minh et al. (2011) studied the impact of cultivation pH on *B. subtilis* spore resistance to heat and high pressure. They applied pH values of pH 6.0 and 10.0. Most of the resulting spores were more resistant than the reference spores produced at optimum conditions What remains open, however, is whether a correlation exists between spore surface hydrophobicity and spore resistance.

Commercially available BI from various suppliers can vary considerably regarding resistance against the respective chemical or physical inactivation process, where they are used to validate the efficiency of inactivation processes. Surface hydrophobicity or the culturing conditions, however, are not among the specified characteristics of commercial spore BI. The hypothesis of Pruß et al. (2012) was that surface hydrophobicity of bacterial spores might possibly influence decontamination with gaseous H_2_O_2_, especially when conditions allow for condensation or micro-condensation. If this was the case, validation of decontamination processes using BI would be affected as well. For hydrophilic BI, inactivation kinetics could lead to false positive results. The decontamination process might be too mild and decontamination not successful. Therefore, it is important to understand the potential impact of cultivation conditions on surface hydrophobicity in order to achieve reproducible validation results.

It is therefore the aim of this study to examine the impact of cultivation pH on surface hydrophobicity and resistance of *B.* *subtilis* spores. Spores were cultivated in a bioreactor with controlled air supply and static cultivation pH levels. Sporulation was induced by exhaustion of the carbon source. The surface of the resulting spores was analyzed by water contact angle measurement. In a first approach the BI resistance was assessed in liquid H_2_O_2_ using a standard protocol also applied under practical conditions and circumstances to evaluate whether an effect can be determined, even when gaseous H_2_O_2_ is finally applied.

## Materials and methods

### Microorganisms

The experiments were carried out with *Bacillus subtilis* (*B. subtilis*, DSM 4181) spores. A freeze dried culture of *B. subtilis* was obtained from the DSMZ (German collection of microorganisms and cell cultures, Braunschweig, DE). The microorganisms were at first revitalized following the manufacturer’s instructions and cultivated on Nutrient Agar (for 1000 mL distilled water: 5.0 g peptone (Merck, Darmstadt, DE), 3.0 g beef extract (Gerbu, Heidelberg, DE), 15 g agar–agar, (Fisher Scientific, Schwerte, DE) with 20 mg of manganese sulfate (Merck, Darmstadt, DE) for 10 days at 37 °C. The spores were subsequently removed from the agar plates by pouring 10 mL of cold, sterile distilled water on the plates, suspending the spores with a spatula and collecting the suspension as starting material for the submersed production of spores in a stirred bioreactor.

### Washing steps

To obtain only the spores the vegetative bacteria were separated by centrifugation at 4000×*g* (10 min, 4 °C) applying a fourfold washing by sterile, distilled water. The spores were heat activated with a temperature of 80 °C for 20 min and subsequently cooled down in ice water. Thereby all remaining vegetative cells were inactivated and simultaneously spores were activated to germinate faster under suitable conditions (Keynan et al. 1964). The targeted purity of the spores was more than 95%, the absence of vegetative cells was checked with a light microscope (Axioskop, Carl Zeiss, Oberkochen, DE). Storage was in distilled water at 4 °C.

### Preparation of inoculum for submersed cultivation

The inoculum was prepared using 100 mL of a sporulation medium (for 1000 mL of distilled water: 5.0 g peptone from casein (Gerbu, Heidelberg, DE), 3.0 g beef extract (Gerbu, Heidelberg, DE), 3.5 g potassium chloride (Merck, Darmstadt, DE), 250 mg magnesium sulphate (Roth, Karlsruhe, DE). After autoclaving 10 mL of 10% glucose in water (Merck, Darmstadt, DE) and 1 mL of the following sterile filtrated micronutrients were added: 1 M calcium nitrate (Sigma Aldrich, Darmstadt, DE) 0.01 M manganese chloride (Merck, Darmstadt, DE), 1 mM iron sulphate (Fluka, Seelze, DE). The medium of pH 7.5 was filled in a 250 mL baffled flask (Duran, Wertheim/Main, DE) and 100 µL of the original spore suspension (5 × 10^8^ cfu/mL) were added. Cultivation took place at 37 °C for 12 h before transferring the bacilli into the bioreactor. The procedure of inoculum preparation was the same for each fermentation.

### Cultivation in the bioreactor

By inoculation of the preculture in 1500 mL of the same medium in a bioreactor (Biostat A plus, Sartorius AG, Göttingen, DE) cultivation was started at 37 °C and constant oxygen supply of 2 L/min filtered air. The pH was kept constant by automatic addition of 0.5 M NaOH (AppliChem, Darmstadt, DE) or 0.5 M HCl (Sigma Aldrich, Darmstadt, DE) with a control unit (Biostat A plus DCU, Sartorius AG, Göttingen, DE). The pH levels were varied between 7.00 and 9.00 in steps of pH 0.50. After 48 h the fermentation was stopped, microorganisms harvested and washed as described above. All spores were subsequently heat activated at 80 °C for 20 min. The viable amount of spores was examined by serial decimal dilutions in Ringer’s solution (Merck, Darmstadt, DE). 100 µL of the appropriate dilutions were plated on plate count agar (for 1000 mL:5.0 g peptone from caseine (Gerbu, Heidelberg, DE), 2.5 g yeast extract (Sigma Aldrich, Darmstadt, DE), 1.0 g glucose (Merck, Darmstadt, DE), 15 g agar–agar (Fisher Scientific, Schwerte, DE)) and incubated at 30 ° for 24 h.

### Contact angle measurement

The surface hydrophobicity was investigated by water contact angle measurement as described in Eschlbeck and Kulozik (2017). In short, filtration of cells was applied (approx. 4 × 10^9^ cells per filter) on a cellulose acetate filter (pore size 0.22 µm, Sartorius Stedim, Göttingen, DE), followed by adjusting the surface water content by storing the filters with spore layers on petri dishes consisting of 2% agar–agar for 2 h to equilibrate the moisture content throughout the spore layer. The filter sheet was cut in stripes and fixed on glass slides by means of double-sided adhesive tape and dried for further 50 min on air to remove excess water and to reach a defined state in moisture content without crack formation of the spore layer. The measurement was carried at out 20 °C with a DSA 100 (Krüss, Hamburg, DE) and the software DSA 4 (Krüss, Hamburg, DE). A drop of water was deposited on the filter surface and the cell hydrophobicity was determined by video analysis of the advancing contact angle.

### Resistance against liquid H_2_O_2_

The test procedure is a modified version of the method described by Muranyi et al. (2006) and Deinhard et al. (2016). Spore suspensions (approx. 10^8^ cfu/mL) were mixed with 35% H_2_O_2_ (Evonik, Essen, DE) at 25 °C at a ratio of 1:99. A magnetic stirrer (200 rpm) was applied for homogeneous spore distribution. Aliquots of 0.1 mL were taken after certain inactivation times and dilution series were generated. The contained H_2_O_2_ was immediately decomposed by preparing the first test tube with 9.8 mL of Ringer’s solution and 0.1 mL of 10% catalase (Catalase from *Micrococcus* *lysodeikticus*, Sigma Aldrich, Darmstadt, DE). Preparing serial decimal dilutions and plating every dilution on two independent plate count agar plates (for 1000 mL: 5.0 g peptone from caseine (Gerbu, Heidelberg, DE), 2.5 g yeast extract (Sigma Aldrich, Darmstadt, DE), 1.0 g glucose (Merck, Darmstadt, DE), 15 g agar–agar (Fisher Scientific, Schwerte, DE)) was followed by incubation for 48 h at 30 °C and detection of the number of survivors.

The aim was to compare the resistance of the spores, therefore a value for resistance is necessary. We chose 1st order reaction kinetic as model for the inactivation curve. With Eq. (1), the resulting decimal reduction time can be calculated for the resulting spores from every cultivation pH. The D value is the time required to inactivate 90% of spores at the given parameters and thus is a measure of resistance of spores against H_2_O_2_ that offers the possibility to compare the resistance properties. In this study, the D value achieved at 25 °C with 35% H_2_O_2_ will be referred to as D_H2O2_. Resistance test was done in triplicate for every spore suspension.

Calculation of the D_H2O2_ was done as shown in Eq. (1).

1 $$ log_{10} S\left( t \right) = - \frac{t}{D} $$

with S being the survival rate at a certain exposition time (t).

## Results

### Concentration of spores

**Fig. 1:**
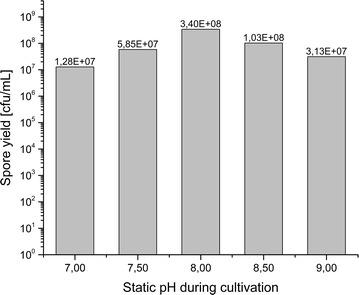
Spore yield shown as colony forming units (cfu) per mL

Submerged bioreactor cultivation resulted in an amout of spores of 1.28 × 10^7^–3.40 × 10^8^ cfu per mL after heat activation as shown in Fig. 1. The highest concentration of spores was obtained at a cultivation pH of 8.00. However, all cultivation pH values yielded amounts of spores only differing slightly more than one log. Cultivation at a static pH of 7.00 and 9.00 can thus be considered successful even with a lower spore yield compared to the other cultivation pH values. The resulting amount of spores were sufficient for water contact angle measurement in triplicate. Higher cultivation pH levels could not be investigated as cultivation at pH 9.50 and 6.50 did not result in a sufficient amount of spores (data not shown).

### Surface hydrophobicity of the spores

**Fig. 2:**
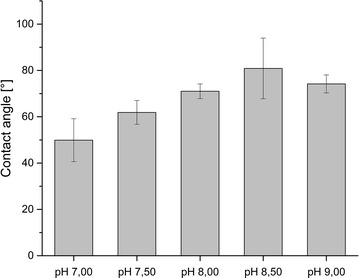
Resulting water contact angles of *B. subtilis* spores cultivated at different pH values

Figure 2 depicts the resulting water contact angles (CA). A trend of increasing contact angles can be seen beginning at pH 7.00 (CA 50°) to a maximum of 80° at pH 8.50. This means that spores become more hydrophobic with increasing pH-value. At a pH of 9.00 a tendency to lower contact angles was found (Fig. 2).

### Resistance towards liquid H_2_O_2_

**Fig. 3:**
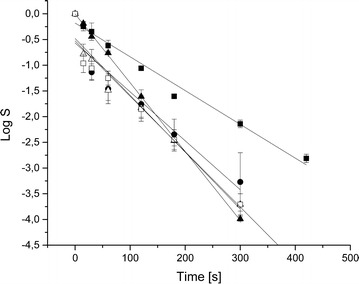
Log linear inactivation trends of the spores cultivated at different pH values (*filled square* pH 7.00, *filled circle* pH 7.50, *filled triangle* pH 8.00, *open triangle* pH 8.50, *open square* pH 9.00)

A higher decimal reduction time means higher resistance towards H_2_O_2_. The resistance towards liquid, 35% H_2_O_2_ is shown as logarithmic survival rate over treatment time in Fig. 3. During the first 15 s of inactivation time, the resulting number of survivors decreases rapidly for spores cultivated at pH 7.50, 8.50 and 9.00. This might be due to an equilibration of cultivation conditions meaning that the least resistant spores of the spore population are quickly inactivated. After the first 15 s log-linear characteristics are present for all spore suspensions.

**Fig. 4:**
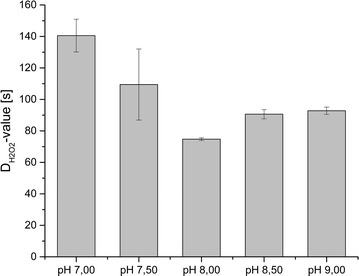
Resulting D_H2O2_-values of the different static pH cultivations, D_H2O2_-values in seconds

The correlation coefficient (R^2^) was calculated for all inactivation curves. For spores cultivated at pH 7.50 R^2^ was 0.89, for all other inactivation curves R^2^ was higher than 0.94. ${\text{D}}_{{{\text{H}}_{ 2} {\text{O}}_{ 2} }}$-values were calculated for each cultivation pH. The D_H2O2_-values are depicted in Fig. 4.

Resistance towards liquid H_2_O_2_ clearly differs depending on cultivation pH. Spores cultivated at pH 8.00 are least resistant whereat a cultivation pH of 7.00 provides spores with the highest resistance.

### Correlation of surface hydrophobicity and resistance

**Fig. 5:**
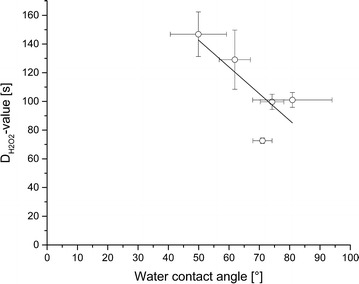
Resistance against liquid H_2_O_2_, shown as D_H2O2_-value in seconds, as a function of the water contact angle with linear regression

To investigate a possible correlation of surface hydrophobicity and resistance as shown in Fig. 5, the resistance against liquid H_2_O_2_ expressed as ${\text{D}}_{{{\text{H}}_{ 2} {\text{O}}_{ 2} }}$-value is plotted as a function of the water contact angle. A linear regression was carried out and the Pearson correlation coefficient was calculated. The correlation coefficient is −0,78 which indicates a negative linear relationship meaning that rising ${\text{D}}_{{{\text{H}}_{ 2} {\text{O}}_{ 2} }}$-values result in lower water contact angles and vice versa.

## Discussion

The obtained amount of spores are in a range of 1.28 × 10^7^–3.40 × 10^8^ cfu per mL and thus slightly dependent on cultivation pH. For the applied cultivation conditions including aeration, media composition and cultivation time, a pH level of 8.00 resulted in a maximum amount of spores. pH-values from 7.00 to 9.00 resulted in similarily high spore yields and provided enough spores for all further experiments. Further optimization of the cultivation conditions will presumably result in higher spore yields, however this was not the aim of the study.

The water contact angles of *B.* *subtilis* spores differ significantly depending on cultivation pH. According to the classification of Rijnaarts et al. (1993), all of the resulting *B. subtilis* spores are hydrophobic, but the degree of hydrophobicity varies.

*Bacillus* *subtilis* spores are surrounded by the outer spore coat consisting of four distinct layers whereat the crust is the outermost one (Imamura et al. 2010; McKenney et al. 2010). Therefore, the crust is the interface between spore and environment and responsible for surface characteristics such as hydrophobicity and adhesion. Some of the proteins localized on the crust surface, e.g. CgeA, CotY and CotZ, have already been identified (Imamura et al. 2011). Chen et al. (2010) applied infrared spectroscopy to identify various functional groups apart from proteins and considered aldehyde and carboxylic acid groups to be responsible for spore adhesion properties. McKenney et al. (2012) state that diversity of the coat structure including the crust is driven by adaption to different niches in nature to survive. Therefore, we suppose that spores tend to adapt to different cultivation pH and that presumably the composition of the crust changes depending on cultivation conditions. This assumption has to be clarified in further studies, however, this study has shown that surface hydrophobicity can be modified by means of various static pH levels.

Our results show that not only surface characteristics but also resistance of *B. subtilis* spores against liquid, 35% H_2_O_2_ varies with the cultivation pH. The ${\text{D}}_{{{\text{H}}_{ 2} {\text{O}}_{ 2} }}$-values of 75–141 s are similar to the ${\text{D}}_{{{\text{H}}_{ 2} {\text{O}}_{ 2} }}$-value of 168 s obtained by Pruß et al. (2012) with a similar method. As Pruß et al. (2012) applied a lower concentration of liquid H_2_O_2_, their time to inactivate 90% of the spore population is slightly longer but in a similar dimension.

Hydrogen peroxide inactivates spores as it damages some component that is needed for the core to expand due to hydration. One reported explanation for variable resistance properties is a change in peptidoglycan composition so that the spore does not expand sufficiently. Another possibility is a quantitative change or altered sensitivity of one or more proteins either needed for remodeling of the expanding cell wall or essential for core expansion itself (Melly et al. 2002b). However, it is not the purpose of this study to clarify the molecular mechanisms, but to approach an aspect of BI application.

A Pearson correlation factor of −0.78 indicates a strong negative correlation between water contact angle and resistance towards liquid hydrogen peroxide. However, surface hydrophobicity measured by contact angle measurement depends on the molecular structure of the outermost layer, the crust, whereat resistance towards liquid hydrogen peroxide presumably depends on some components inside the spore. Therefore, this correlation factor seems quite high and a possible correlation between cell surface hydrophobicity and resistance has to be verified in further experiments.

Surface hydrophobicity is suspected to have an impact on the decontamination of surfaces such as packaging material when gaseous, condensing hydrogen peroxide is applied. Surface hydrophobicity as well as resistance towards liquid H_2_O_2_ depend on cultivation pH. At the moment, no standardized protocol for the cultivation of BI exists. Our results show clearly that a standardization is very important as cultivation pH does not only influence spore resistance but also spore characteristics that are not screened on a regular base such as surface hydrophobicity. The development of a standardized production and application of BI should result in more consistent inactivation test results.

## Abbreviations

BI: biological indicator
*B.* *subtilis*: *Bacillus* *subtilis*
D_H2O2_: decimal reduction time of spores at 25 °C and 35% H_2_O_2_
H_2_O_2_: hydrogen peroxide
R^2^: correlation coefficient
